# The roles and potential mechanisms of plant polysaccharides in liver diseases: a review

**DOI:** 10.3389/fphar.2024.1400958

**Published:** 2024-06-20

**Authors:** Xianzhi Wei, Daimin Luo, Haonan Li, Yagang Li, Shizhuo Cen, Min Huang, Xianxing Jiang, Guoping Zhong, Weiwei Zeng

**Affiliations:** ^1^ School of Pharmaceutical Sciences, Sun Yat-sen University, Guangzhou, China; ^2^ Guangdong Provincial Key Laboratory of New Drug Design and Evaluation, Guangzhou, China; ^3^ Shenzhen Longgang Second People’s Hospital, Shenzhen, China

**Keywords:** plant polysaccharides, anti-inflammation, antifibrotic, non-alcoholic fatty liver disease, alcohol-related liver disease, drug-induced liver injury, hepatocellular carcinoma

## Abstract

Plant polysaccharides (PP) demonstrate a diverse array of biological and pharmacological properties. This comprehensive review aims to compile and present the multifaceted roles and underlying mechanisms of plant polysaccharides in various liver diseases. These diseases include non-alcoholic fatty liver disease (NAFLD), alcoholic liver disease (ALD), fibrosis, drug-induced liver injury (DILI), and hepatocellular carcinoma (HCC). This study aims to elucidate the intricate mechanisms and therapeutic potential of plant polysaccharides, shedding light on their significance and potential applications in the management and potential prevention of these liver conditions. An exhaustive literature search was conducted for this study, utilizing prominent databases such as PubMed, Web of Science, and CNKI. The search criteria focused on the formula “(plant polysaccharides liver disease) NOT (review)” was employed to ensure the inclusion of original research articles up to the year 2023. Relevant literature was extracted and analyzed from these databases. Plant polysaccharides exhibit promising pharmacological properties, particularly in the regulation of glucose and lipid metabolism and their anti-inflammatory and immunomodulatory effects. The ongoing progress of studies on the molecular mechanisms associated with polysaccharides will offer novel therapeutic strategies for the treatment of chronic liver diseases (CLDs).

## Graphical Abstract

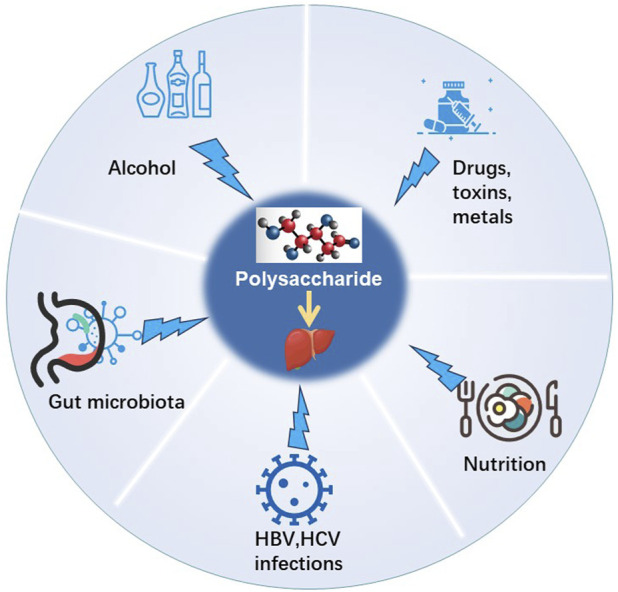

## 1 Introduction

Chronic liver disease (CLD) has emerged as a consequential global public health challenge, significantly contributing to morbidity and mortality rates worldwide. In recent decades, the prevalence of liver diseases has been steadily escalating, establishing them as leading causes of death and illness on a global scale. These diseases, encompassing cirrhosis, viral hepatitis, and liver cancer, are responsible for an annual loss of over two million lives, accounting for approximately 4% of all worldwide deaths (equating to 1 out of every 25 deaths). Notably, liver cancer alone contributes to a staggering 600,000 to 900,000 fatalities. Presently, liver disease ranks as the eleventh-leading cause of death, although the actual number of liver-related deaths may be underestimated. A current study indicates that cirrhosis ranks as the tenth-leading cause of death in Africa (thirteenth-leading cause in 2015), the ninth-leading cause in Southeast Asia and Europe, and the fifth-leading cause of death in the Eastern Mediterranean region (Devarbhavi et al., 2023). Notably, the incidence of viral hepatitis has shown a decline in most countries due to progress in disease prevention, diagnosis, and therapeutic interventions. Moreover, the implementation of comprehensive immunization programs targeting the hepatitis B virus has proven effective in reducing the number of new cases in numerous countries (Xiao et al., 2019). With the advancement in living standards, there is an anticipated increase in the prevalence of metabolic liver diseases, namely non-alcoholic fatty liver disease (NAFLD), alcohol-related liver disease (ALD), and drug-induced liver injury (DILI). Consequently, this rise in cases is expected to result in an escalation of end-stage liver diseases, including liver failure, cirrhosis, and liver cancer. As a direct consequence, liver diseases unequivocally emerge as significant contributors to morbidity and mortality rates within the whole world.

Newly discovered elements, such as stem cells and miRNAs targeting specific genes, have emerged as potential novel mechanisms contributing to CLD. These discoveries have significantly influenced liver disease research in the past decade. However, it is important to note that most liver diseases do not have a single cause, but rather multiple concurrent causes. For instance, individuals may experience infection of hepatitis B virus superimposed on metabolic dysfunction–associated steatotic liver disease (MASLD) (Rinella et al., 2023). The molecular mechanisms of liver disease primarily involve synergistic effects and multi-target signaling pathways, rather than relying solely on single targets or signaling pathways. Consequently, the use of a single drug to treat a specific disease inherently presents limitations, as compared to the approach of employing a multi-level, multi-target, and multi-signaling strategy. Future therapeutic interventions aimed at improving liver disease outcomes are anticipated to employ rational structural optimization and design strategies based on the understanding of structure-activity relationships. These interventions will target the modulation of well-established signaling pathways, which hold significant importance in the context of multifactorial liver diseases, to ameliorate CLD. However, the inadequate knowledge regarding the pathogenesis of liver diseases, coupled with delayed diagnoses and rapid disease progression, contribute to the insufficiency of current clinical therapeutic approaches. These limitations directly result in unsatisfactory treatment outcomes.

Natural polysaccharides possess distinctive structural characteristics that encompass factors such as molecular weight, monosaccharide composition, charge properties, and glycosidic bonds. These features not only determine the functional attributes of polysaccharides but also contribute to their extensive utilization in various applications. PP exhibit diverse biological activities and hold tremendous potential in mitigating liver damage caused by conditions such as NAFLD, ALD, DILI, hepatic fibrosis, and HCC.

Plant polysaccharides hold potential as valuable sources of therapeutic agents for liver disease due to their low toxicity and ability to target multiple processes and pathways. However, the growing number of studies investigating the effective plant-derived compounds have yet to be systematically summarized, particularly with regards to the functions and mechanisms of plant polysaccharides exhibiting hepatoprotective effects. Therefore, this comprehensive review aims to address this gap by specifically focusing on the mechanisms underlying the actions of polysaccharides in liver disease therapy.

## 2 Methods

The research conducted for this study involved a meticulous search of prominent online academic databases, including PubMed, Web of Science, and CNKI, up until the year 2023. Our search strategy utilized specific terms such as “plant polysaccharides” and “liver disease”, as well as various combinations of these terms. Within the timeframe of 2010–2023, more than 140 scholarly articles were identified, focusing on the potential of plant polysaccharides in addressing liver conditions such as NAFLD, ALD, DILI, hepatic fibrosis, and HCC. These articles were systematically categorized based on their primary objectives, and their key findings were summarized for clarity. Additionally, to ensure a comprehensive understanding and provide historical context, a thorough review of influential studies published prior to 2010 was also conducted. This approach ensures a holistic perspective on the topic, encompassing both recent advancements and foundational knowledge.

### 2.1 Polysaccharides in different liver diseases

#### 2.1.1 Plant polysaccharides against non-alcoholic fatty liver disease

NAFLD represents the hepatic manifestation of a cluster of conditions linked to metabolic dysfunction. The prevalence of NAFLD on a global scale tends to go up (Friedman et al., 2018). Globally, the estimated prevalence of NAFLD stands at approximately 25%, with the highest rates observed in the Middle East and South America, and the lowest in Africa. In North America and Europe, NAFLD is commonly associated with central obesity, accounting for approximately 83% of affected patients. However, it is noteworthy that in Asia, a significant proportion of NAFLD patients, known as “thin NASH” individuals, exhibit normal body mass index (BMI), despite the lower BMI threshold for defining overweight in Asia (BMI > 23) compared to North America and Europe (BMI > 25) (Pereira et al., 2015). NAFLD is characterized by the accumulation of fat (steatosis) in more than 5% of hepatocytes, occurring concurrently with metabolic risk factors, particularly obesity and type 2 diabetes. Notably, NAFLD is distinguished by the absence of excessive alcohol intake (≥30 g per day for men and ≥20 g per day for women), as well as the absence of other chronic liver diseases (Byrne and Targher, 2016). NAFLD encompasses a spectrum that spans from isolated steatosis, in which fat accumulates in the liver without significant progression, to the more severe condition known as non-alcoholic steatohepatitis (NASH). NASH is distinguished by the presence of hepatocellular injury, inflammation, and fibrosis, and is characterized by a progressive clinical course. Left untreated, NASH may lead to the development of cirrhosis, with its associated complications including hepatocellular carcinoma.

**FIGURE 1:**
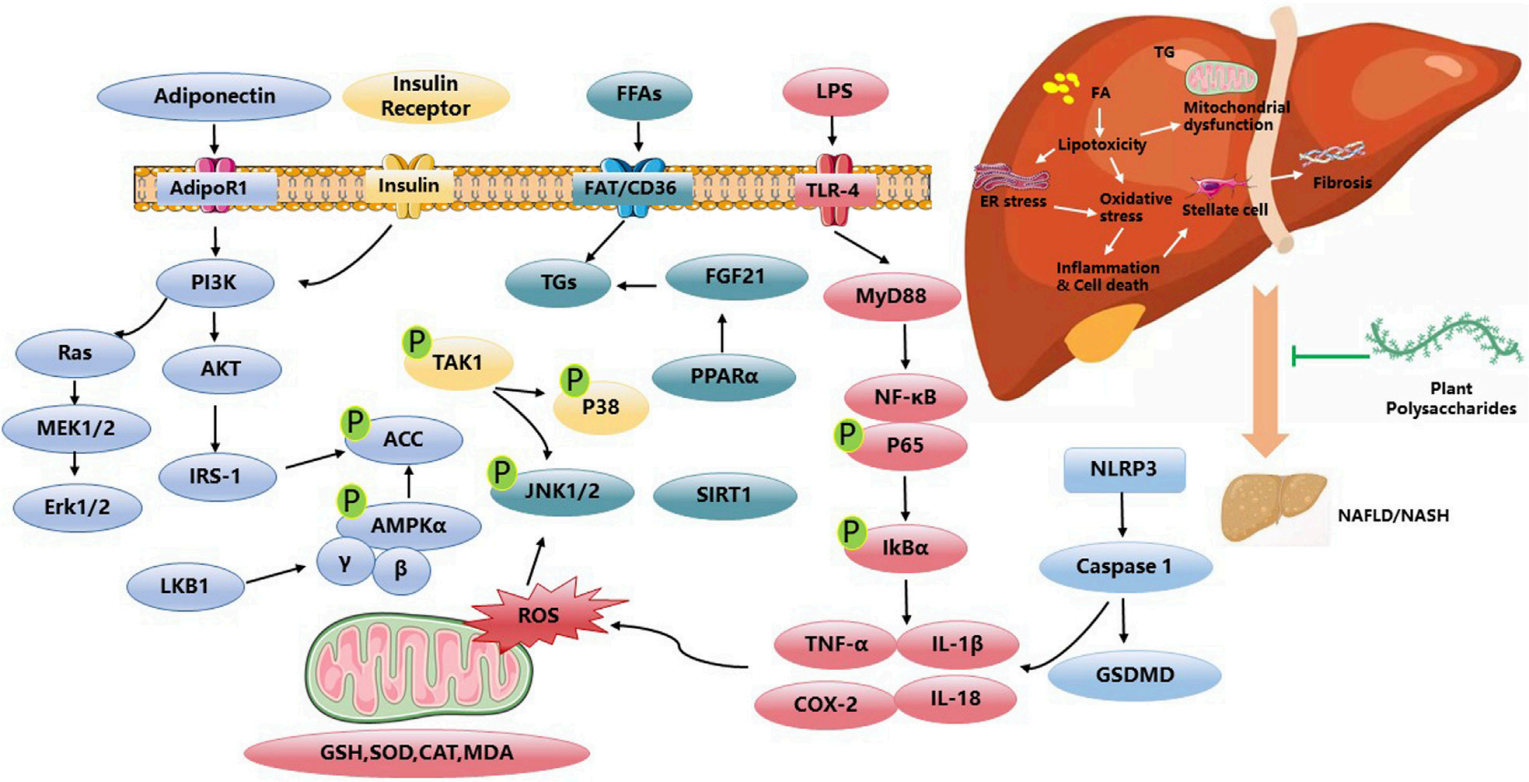
Anti-NAFLD activities and related mechanisms of PP.

PP have shown the potential to alleviate the effects of NAFLD by inhibiting hepatocellular injury, inflammation, and fibrosis. The underlying mechanism is closely associated with the regulation of energy metabolism mediated through signaling pathways such as AMPK and MAPK. Radix *Hedysari* polysaccharide, *Polygonatum sibiricum* polysaccharides, and *Astragalus mongholicus* polysaccharides have been reported to ameliorate disorders in lipid metabolism, regulate hepatic lipid content, and improve liver inflammation and damage by modulating the phosphorylation levels of AMPK (Sun et al., 2014; Huang et al., 2021; Zhou et al., 2021; Zhong et al., 2022). Up to now, APS has been widely used in poultry and animal feed, with the function of improving the utilization of nutrients and promoting animal growth. The application of APS in the human is mainly to prevent and treat cardiovascular diseases. Additionally, the MAPK cascade plays a role in regulating NF-κB gene expression through redox mechanisms. ASP has been shown to mitigate Caspase-3-dependent apoptosis through the involvement of the Caspase-8 and JNK-mediated pathway. Moreover, ASP inhibits the activation of IL-6/STAT3 and NF-κB signaling pathways (Wang et al., 2016). Furthermore, emerging evidence supports the significant involvement of the gut microbiota in the development and progression of NAFLD. This study contributes novel findings by demonstrating the potential of *Astragalus mongholicus* polysaccharides to alleviate hepatic inflammation and reduce lipid accumulation in NAFLD. These beneficial effects are achieved through the modulation of the gut microbiota composition and the SCFA-GPR signaling pathways (Zhong et al., 2022). Moreover, *Poria cocos* polysaccharides have demonstrated the potential to mitigate the disruption of the gut–vascular barrier, the translocation of endotoxins induced by a high-fat diet, and inhibit intestinal pyroptosis. These effects are mediated through the regulation of key factors, including PARP-1 and the administration of pyroptosis inhibitors, such as MCC950 (Ye et al., 2022). Walnut green husk polysaccharide has the potential to enhance the composition and diversity of the gut microbiota, as well as increase the abundance of beneficial bacteria (Wang et al., 2020). Long-term and repetitive inflammation is a contributing factor in the advancement of NAFLD. In the progression of NAFLD, inflammation, fibrosis, autophagy, and apoptosis interact and exacerbate one another. *Gynostemma pentaphyllum* polysaccharides have been shown to inhibit the expression of Toll-like receptor 2 (TLR2) and downregulate the expression of the NOD-like receptor pyrin domain-containing 3 (NLRP3) inflammasome, as well as the pro-inflammatory cytokines TNF-α and IL-1β. These polysaccharides have the potential to improve non-alcoholic steatohepatitis (NASH), possibly through the modulation of gut microbiota and the TLR2/NLRP3 signaling pathway (Yue et al., 2022). *Angelica sinensis* polysaccharide (ASP) has garnered significant attention due to its notable hepatoprotective effects. Previous research has demonstrated that ASP exerts therapeutic effects on NAFLD through the regulation of lipid metabolism via the propionate/ERRα pathway (Luo et al., 2023). Furthermore, ASP has the capacity to enhance the expression of PPARγ and key liver insulin signaling proteins, such as IRS-2, PI3K, Akt, p-Akt, and GLUT2. Moreover, ASP has been shown to increase the levels of the anti-apoptotic protein Bcl-2 while concurrently reducing the expression of the pro-apoptotic protein Bax. This multifaceted action of ASP not only provides protection against hepatic damage but also offers promising therapeutic benefits in the context of liver health (Wang et al., 2016). *Gynostemma pentaphyllum* polysaccharides is a pure natural plant with medicinal value, which has broad application prospects in food, health, and drug. At present, the utilization is only for crude products, and research on it is limited to preclinical studies. Various studies have been done for discovering the anti-NAFLD activity of plant polysaccharides in Figure 1. Table 1 gives an overview of some studies performed in NAFLD.

**TABLE 1:** Summary of known anti-NAFLD activity of PP.

Polysaccharide	Origin plant	Models	Functions	Mechanisms	References
Radix *Hedysari* polysaccharide	Radix *Hedysari*	HFD rat	Ameliorate lipid metabolism disorders	Activate P-AMPK, pparα and downregulate SREBP-1c	Sun et al. (2014)
*Ginkgo biloba* polysaccharide	*Ginkgo biloba*	HFD rat	Enhance antioxidant defence system	Reduce lipid peroxidation	Yan et al. (2015)
*Angelica sinensis* polysaccharide	*Angelica sinensis* (Oliv.) Diels	ConA-induced liver damage in mice	Anti-inflammatory and anti-oxidant	Attenuate Caspase-3-dependent apoptosis by Caspase-8 and JNK-mediated pathway	Wang et al. (2016b)
*Sophora flavescens* polysaccharides	*Sophora flavescens*	ConA-induced hepatitis mice	Anti-inflammatory and anti-oxidant	Inhibit activation of NKT cell, inhibit HBV secretion	Yang et al. (2022)
Enteromorpha prolifera polysaccharide	Enteromorpha prolifera	HFD rat	Upregulate cystathionine-β-synthase	Increase H2S production	Ren et al. (2020)
*Artemisia sphaerocephala* Krasch polysaccharide	*Artemisia sphaerocephala* Krash seed	HF mice	Maintenance of the intestinal microecosystem	Modulate hepatic SREBP-1c, SCD-1, ACC and FAS expression	Zhang et al. (2019)
Trehalose	The ergot of rye	HFD mice	The induction of autophagy	Inhibit atherosclerosis and attenuate hepatic steatosis	Stachowicz et al., (2019)
Codonopsis lanceolata polysaccharide	Codonopsis lanceolata	HFHS mice	Ameliorate insulin resistance	Impair phosphorylation of PKB/Akt and hyperphosphorylation of IRS-1 at Ser307	Zhang et al. (2019)
Walnut green husk polysaccharide	Walnut green husk	HF mice	Ameliorate oxidative stress, lipid metabolism	Improve the composition of gut microbiota, increase the diversity of gut microbiota and the abundance of beneficial bacteria	Wang et al. (2020)
Noni fruit polysaccharide	Noni fruit	HFD rat	Improve hepatic oxidative stress and inflammation	Modulate short-chain fatty acids (SCFAs), the intestinal barrier, and gut microbiota	Yang et al. (2022)
*Polygonatum sibiricum* polysaccharides	*Polygonatum sibiricum*	HFD rat	Promote lipid metabolism, decrease body weight, and anti-inflammatory and anti-oxidant	Upregulate insulin receptor expression, increase AMPK phosphorylation, and downregulate SREBP2 and LDLR expression	Huang et al. (2021)
*Poria cocos* polysaccharides	*Poria cocos*	NAFLD mice	Inhibit pyroptosis	Inhibit the pyroptosis of small intestinal macrophages	Ye et al. (2022)
*Astragalus mongholicus* polysaccharides	*Astragalus mongholicus*	NAFLD rat	Ameliorate hepatic lipid accumulation and inflammation as well as modulate gut microbiota	Increase p-AMPK and PPAR-α, decrease SREBP-1, TLR4, NF-κB NLRP3, GPR41 and 43. Modulate the gut microbiota and SCFA-GPR signaling pathways	Zhong et al. (2022)
*Polygonatum cyrtonema* Hua polysaccharides	*Polygonatum cyrtonema* Hua	NAFLD mice	Reduce liver damage, improve lipid metabolism, decrease oxidative stress	Promote the production of short-chain fatty acids, and balances the composition of the intestinal microbiota	Liu et al. (2021)
Pleurotus polysaccharides	Pleurotus	HepG2 cells	Regulate liver-gut axis system	Increase the viabilities and cellular total superoxide dismutase activities	Huang et al. (2021)

#### 2.1.2 Plant polysaccharides against ethanol-induced liver disease

ALD encompasses a range of liver conditions resulting from excessive alcohol consumption. These conditions include liver steatosis, steatohepatitis, hepatitis, cirrhosis, and HCC. The progression of ALD is primarily influenced by the duration and quantity of alcohol intake, while genetic, epigenetic, and environmental factors also contribute. Chronic alcohol use is a prominent cause of morbidity and mortality on a global scale, impacting over 200 disease and injury outcomes (Rehm et al., 2017; European Association for the Study of the Liver, 2018). According to the World Health Organization (WHO), there are approximately 2.3 billion current consumers of alcoholic beverages, with around one billion categorized as heavy intermittent drinkers. Among alcohol-attributable conditions, cirrhosis of the liver obtains the highest score, followed by road injuries and other digestive diseases (Saito et al., 2018). Alcohol is widely acknowledged as a carcinogen, being associated with the development and progression of various types of cancer. Furthermore, alcohol consumption has been firmly linked to the advancement of liver-specific diseases, including chronic viral hepatitis and hepatocellular carcinoma (Dolganiuc, 2015; Sahlman et al., 2016; Ganesan et al., 2020).

The liver assumes primary responsibility for ethanol metabolism. When excessive alcohol is consumed, the liver incurs substantial tissue damage as a result of both oxidative stress and the accumulation of acetaldehyde and lipopolysaccharide (LPS) (Meroni et al., 2018; Kong et al., 2019). ALD encompasses various conditions linked to alcohol consumption, such as early-stage asymptomatic ALD characterized by fatty liver or steatosis, steatohepatitis, advanced forms including alcoholic hepatitis and cirrhosis, as well as the development of HCC(Thursz et al., 2019).

Inflammation is a crucial risk factor associated with the progression of ALD, serving as a prerequisite for the development of fibrosis, cirrhosis, and HCC. Activation of Toll-like receptor 4 (TLR4) triggers NF-κB signaling, leading to the production and release of pro-inflammatory cytokines such as tumor necrosis factor (TNF) and interleukin-6 (IL-6). Chronic alcohol consumption increases the levels of TNF and IL-6 in both animal models and human liver biopsy samples. Notably, patients with acute alcoholic hepatitis exhibit significantly elevated circulating levels of TNF and IL-6, which have been implicated in disease severity and the onset of multiorgan failure (Seitz et al., 2018).

**FIGURE 2:**
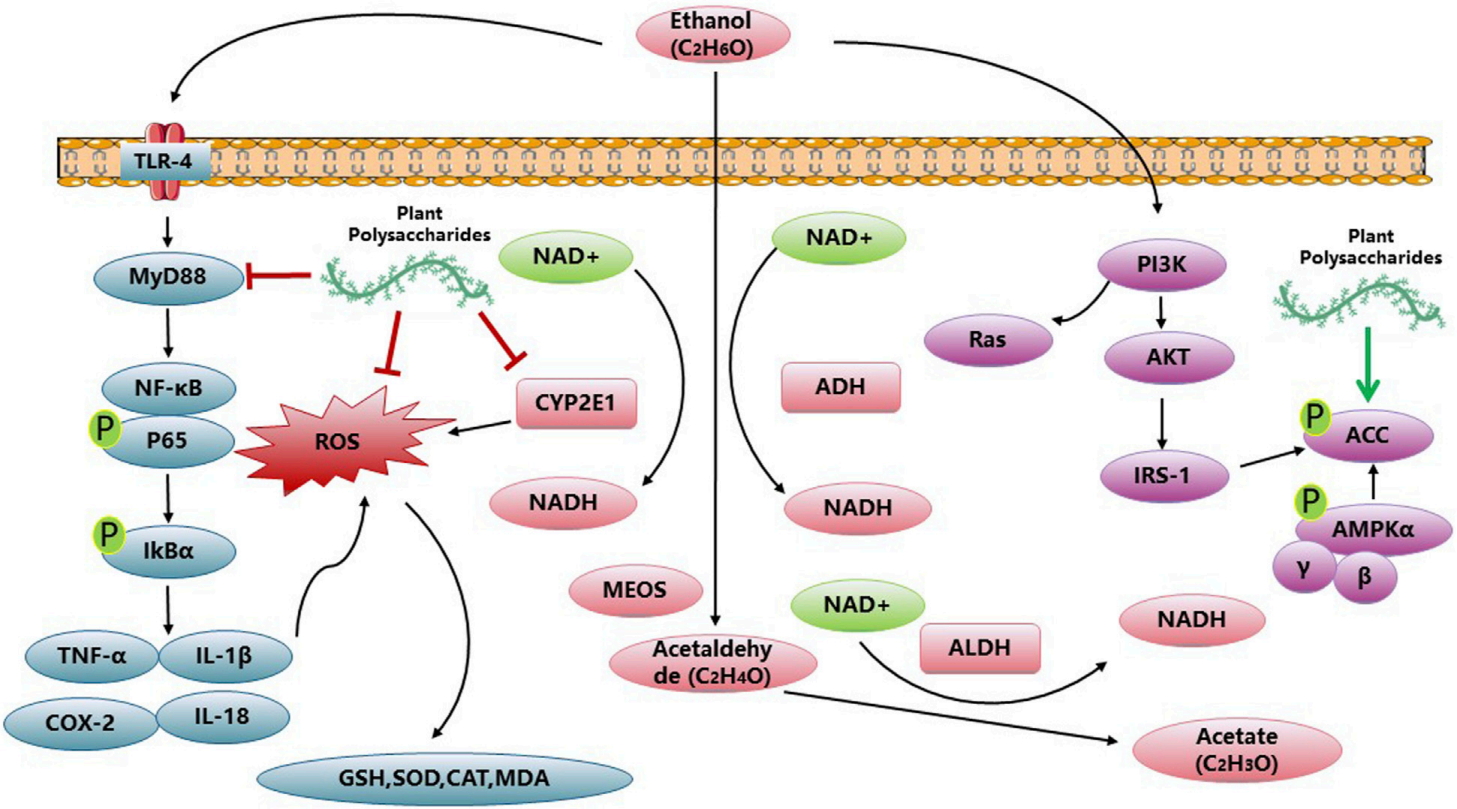
Anti-ALD activities and related mechanisms of PP.

PP exhibit notable anti-inflammatory properties, and recent studies have indicated that *Aloe vera* polysaccharides (AVP) can ameliorate ALD. AVP achieves this by upregulating AMPK-α, PPAR-α, and IκB-α, while simultaneously downregulating TLR-4 and MyD88 (Cui et al., 2014). Additionally, *Aloe vera* polysaccharides have been found to reduce hepatic inflammation by inhibiting the toll-like receptor 4 (TLR4)/nuclear factor-kappa B (NF-κB) signaling pathway. Moreover, they improve hepatocyte apoptosis by inhibiting the CYP2E1/ROS/MAPKs signaling pathway (Jiang et al., 2022). *Lycium barbarum* polysaccharide was found to regulate the NLRP3 inflammasome pathway, effectively inhibiting hepatic inflammation in the context of ALD. Moreover, it was observed that *Lycium barbarum* polysaccharide primarily ameliorated ALD through the SCD1-AMPK-CPT pathway, subsequent to ERα (Xiao et al., 2014; Wang et al., 2018). The activation of PPAR-γ signaling by water-insoluble polysaccharide treatment effectively reduces inflammation in colonic epithelial cells and promotes a hypoxic state, which aids in suppressing the excessive growth of fungi and Proteobacteria in the gut. This mechanism holds promise for alleviating ALD (Sun et al., 2020). A polysaccharide known as PFP-1, obtained from the fruiting body of *Pleurotus geesteranus*, has exhibited the ability to mitigate oxidative stress and inflammatory responses. This effect is achieved through the activation of Nrf2-mediated signaling pathways and regulation of the TLR4-mediated NF-κB signaling pathways, presenting a potential therapeutic strategy against ALD (Song et al., 2021). Additionally, research has indicated that the lipid-lowering impact of ASP may stem from its dual inhibition of lipid synthesis and CD36-mediated lipid uptake. The antioxidative properties of ASP can be attributed to its ability to reverse alcohol metabolic pathways, transitioning from cytochrome P450 2E1 (CYP2E1) catalysis to alcohol dehydrogenase (ADH) catalysis. Overall, this study establishes the direct involvement of ASP in lipid metabolism and elucidates its mechanism of action in reducing reactive oxygen species (ROS), thus positioning it as a potential therapeutic agent for the treatment of alcoholic fatty liver disease (AFLD) (He et al., 2022). The polysaccharides derived from *Echinacea purpurea*, known as EPPs, exhibit significant free radical scavenging activity *in vitro*, and have demonstrated the ability to ameliorate alcohol-induced liver injury through the activation of Nrf2/HO-1 pathways *in vivo* (Jiang et al., 2021; Jiang et al., 2022). These findings highlight the remarkable potential of PP in effectively regulating abnormal biochemical indices associated with ALD. Figure 2 and Table 2 give a summary of a few reports that PP against ALD.

**TABLE 2:** Given a summary of a few reports on the anti-ALD activity of PP.

Polysaccharide	Origin plant	Models	Functions	Mechanisms	References
*Aloe vera* polysaccharides	*Aloe vera*	ALD induced-mouse	Anti-oxidation, anti-inflammation and immune enhancement	Upregulate AMPK-α,PPAR-α and IκB-α; downregulation of TLR-4 and MyD88	Cui et al. (2014)
*Lycium barbarum* polysaccharide	*Lycium barbarum*	ALD induced-mice	Rebalance the dysregulated lipid metabolism	Activate the SCD1-AMPK-CPT pathway; TXNIP-NLRP3 inflammasome pathway	Wang et al. (2018), Xiao et al. (2014)
*Crassostrea gigas* water-soluble polysaccharide	*Crassostrea gigas*	ALD induced-mice	Antioxidant	Decrease serum AST, ALT, and MDA; increase SOD	Shi et al. (2015)
*Dendrobium huoshanense* polysaccharide	*Dendrobium huoshanense*	ALD induced-mice	Restore the perturbed metabolism pathways	Alter metabolic levels particularly involved in phosphocholine and l-Proline	Wang et al. (2016)
Mori Fructus polysaccharides	Morus alba L.	ALD induced-rats	Anti-inflammatory antioxidant, and immuno-enhancing activities	Activation of ethanol dehydrogenase, elimination of free radicals, and inhibition of lipid peroxidation	Zhou et al. (2021)
*Triticum aestivum* sprout-derived polysaccharide	ALD induced-mouse	ALD induced-mice	Inhibit steatosis and improve antioxidant marker levels	Regulated by a phosphatidylinositol 3-kinase (PI3K)/Akt pathway	Nepali et al. (2017)
Schisandra chinensis acidic polysaccharide	Schisandra chinensis acidic	ALD induced-mice	Alleviate oxidative stress	Inhibit the upregulation of CYP2E1	Yuan et al. (2018)

#### 2.1.3 Plant polysaccharides against drug-induced liver injury (DILI)

The liver is highly susceptible to drug toxicity during clinical treatment due to its first-pass effect in gastrointestinal nutrition metabolism. Drug-induced liver injury is estimated to affect around 14–19 cases per 100,000 individuals (Andrade et al., 2019). Although asymptomatic elevated liver enzymes are the most common presentation, drug-induced liver injury constitutes the primary cause of acute liver failure in many Western countries, accounting for over 50% of cases. It can manifest at either excessive or therapeutic doses, potentially as consequence of direct intrinsic drug hepatotoxicity or idiosyncratic (unpredictable) hepatotoxicity (Hoofnagle and Bjornsson, 2019).

**FIGURE 3:**
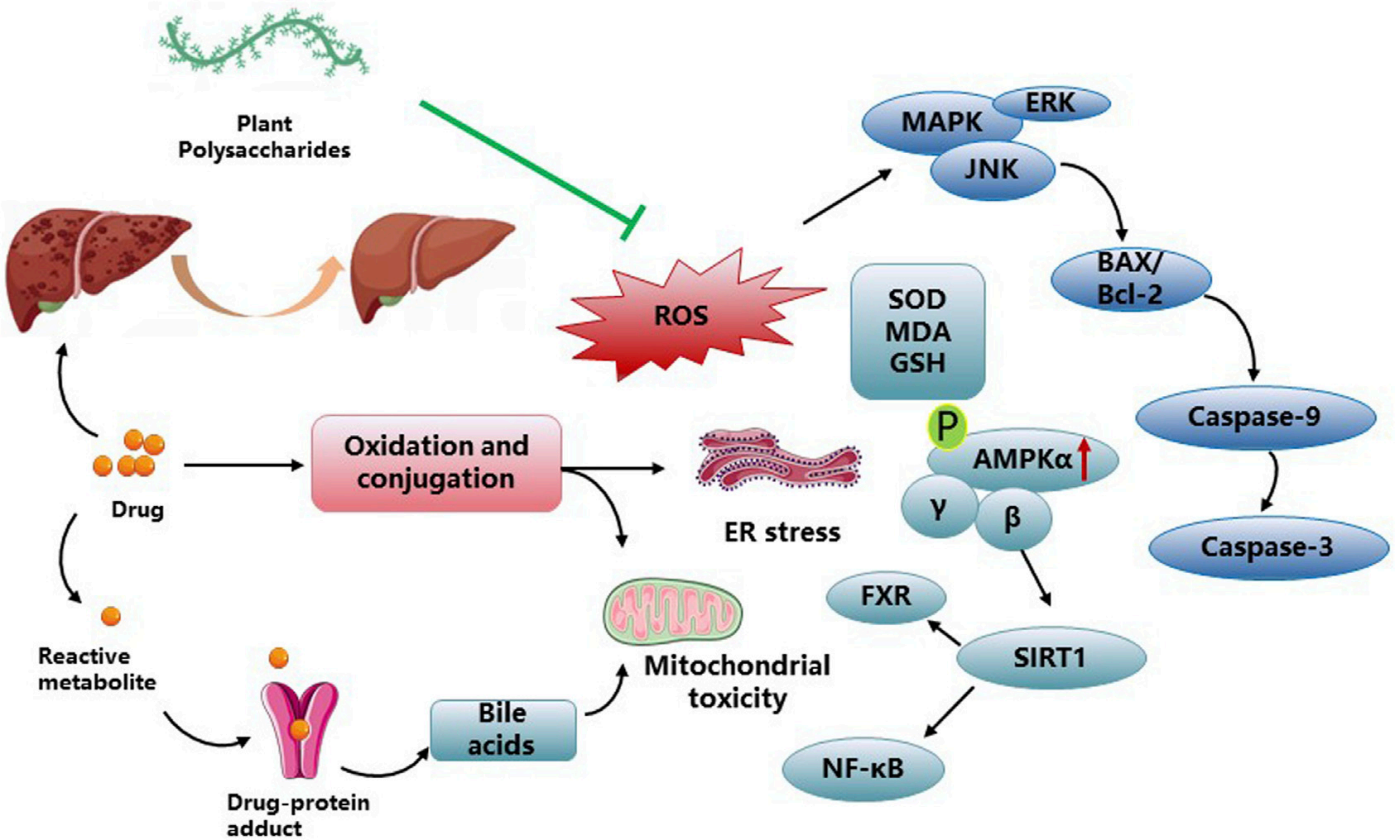
PP impact on DILI activities and related mechanisms.

DILI is an infrequent condition that occurs irrespective of drug dose, route, or duration of administration. Moreover, idiosyncratic DILI does not represent a single homogeneous disease, but rather a range of rare disorders presenting diverse clinical, histological, and laboratory characteristics. The pathogenesis of DILI remains incompletely elucidated, with various factors contributing to its development and progression (Fontana, 2014). Intrinsic hepatotoxins, such as acetaminophen, typically exhibit dose-dependent behavior and can be studied using reproducible animal models to understand the underlying pathways leading to hepatocyte injury. Conversely, most cases of DILI observed in clinical practice are considered “idiosyncrasies” because they lack a clear correlation with the dose, route, or duration of administration, which makes them specific to each patient. PP play a significant protective role in mitigating drug-induced liver damage. In addition to the direct toxic effects of drugs, oxidative stress can occur as a result of drug metabolism, leading to the generation of ROS. These ROS can interact with proteins, causing changes in their functional and structural characteristics, and form neoantigens. ROS are responsible for initiating lipid peroxidation, leading to the formation of lipid peroxidation byproducts, such as 4-hydroxynonenal (4-HNE) and malondialdehyde (MDA). Various studies have demonstrated the potential of PP in improving ROS levels associated with DILI. Notable examples include Jujube polysaccharides, Seabuckthorn berry polysaccharide, and *Periploca* polysaccharide (Liu et al., 2015; Athmouni et al., 2018; Wang et al., 2018). *Periploca* polysaccharides have shown significant reductions in MDA content and protein damage in liver tissue, along with improvements in liver function parameters (alanine transaminase, ALT; aspartate aminotransferase, AST; bilirubin). Furthermore, *Periploca* polysaccharides have demonstrated the ability to enhance the activities of hepatic antioxidant enzymes (superoxide dismutase, SOD; catalase, CAT; glutathione peroxidase, GPx; GSH) as a protective response against cadmium chloride (CdCl_2_)-induced toxicity in male Wistar rats (Athmouni et al., 2018). Schisandra polysaccharide exhibited a significant reduction in ALT, AST, TNF-α, and IL-1β levels, leading to the alleviation of hepatic pathological alterations in the mouse model. Additionally, it demonstrated protective effects on liver injury-associated enzymes and factors, including a notable decrease in MDA levels and GSH depletion, downregulation of Bax/Bcl-2 expression, inhibition of cleaved caspase-3 expression, as well as upregulation of p-AMPK, p-Akt, GSK 3β, Nrf 2, and HO-1 proteins in the liver tissues of the mouse model (Che et al., 2019). The hepatoprotective effect of *Echinacea purpurea* polysaccharide against APAP-induced DILI was observed. This effect was associated with a decrease in autophagy-dependent oxidative stress, inflammation, and apoptosis. Moreover, the observed protective mechanism involved Parkin-dependent autophagy (Yu et al., 2022). ASP pretreatment demonstrated significant attenuation of Caspase-3-dependent apoptosis through the Caspase-8 and JNK-mediated pathway. Furthermore, ASP inhibited the activation of IL-6/STAT3 and NF-κB signaling pathways in ConA-induced liver damage in mice (Wang et al., 2016). Moreover, ASP exhibits potential as a hepatoprotective agent for the management of acetaminophen (APAP)-induced liver injury by increasing glutathione (GSH) levels and inhibiting hepatic apoptosis (Cao et al., 2018). Notably, ASP has also shown efficacy in alleviating chronic liver fibrosis by inhibiting HSC activation via the IL-22/STAT3 pathway (Wang et al., 2020). *Broussonetia papyrifera* polysaccharide demonstrated hepatoprotective properties against APAP-induced liver injury. It effectively attenuated liver apoptosis, enhanced antioxidant capacity, and improved the liver’s detoxification ability towards APAP (Xu et al., 2022). Several studies have been conducted to investigate the efficacy of plant polysaccharides on the treatment of drug-induced liver damage in Figure 3 and Table 3.

**TABLE 3:** Summary of articles data about PP impact on DILI activity.

Polysaccharide	Origin plant	Models	Functions	Mechanisms	References
*Jujube* polysaccharides	*Zizyphus jujube* cv	CCl_4_ or APAP induced in mice	Anti-oxidation and detoxification	Enhance SOD and GSH-Px and decrease MDA	Liu et al. (2015)
*dandelion* root polysaccharide	*dandelion*	APAP induced in mice	Enhance Nrf2, NQO1 and HO-1, decrease Keap1	Activate the Nrf2-Keap1 pathway	Cai et al. (2015)
*Seabuckthorn* berry polysaccharide	*seabuckthorn*	APAP induced in mice	Increase GSH and GSH-Px, SOD and SOD-2; the ratio of Bcl-2/Bax,Nrf-2; reduced NO and iNOS and p-JNK; Keap-1	The activation of the Nrf-2/HO-1-SOD-2 signaling pathway	Wang et al. (2018)
*Periploca* polysaccharide	*Periploca*	Cadmium chloride (CdCl_2_) induced toxicity in male Wistar rats	Antioxidant	Decrease the content of MDA and protein damage, hepatic anti-oxidant	Athmouni et al. (2018)
*Schisandra chinensis* acidic polysaccharide partially	*Schisandra chinensis*	APAP induced in mice	Antioxidation, anti-inflammation and anti-apoptosis	Reduce ratio of Bax/Bcl-2, prohibit cleaved caspase-3, and elevate p-AMPK, p-Akt, p- GSK 3β, Nrf 2 and HO-1	Che et al. (2019)
*Astragalus* polysaccharide	*Astragalus*	Cantharidin (CTD)-induced-mice	Inhibit oxidative stress; regulate primary bile acid biosynthesis and glycerophospholipid metabolism	Inhibit ER stress	Huang et al. (2021)
*Echinacea purpurea* polysaccharide	*Echinacea purpurea*	APAP overdose-induced DILI in mice	Increase autophagy with a reduction in oxidative stress and inflammation	Reduction of autophagy-dependent oxidant, inflammatio-n and apoptosis	Yu et al. (2022)
*Salvia miltiorrhiza* polysaccharides	*Salvia miltiorrhiza*	Florfenicol induced in chickens	Inflammation and oxidative stress	The phagosome signaling pathway	Wang et al. (2022)

#### 2.1.4 Plant polysaccharides against hepatic fibrosis

Hepatic fibrosis and cirrhosis pose a substantial global health burden, leading to liver failure or HCC, thereby posing a significant threat to human health on a worldwide scale (Yang et al., 2023). Liver diseases pose a substantial global threat to human health, contributing to approximately 2 million deaths annually. Hepatic cirrhosis alone accounts for approximately 50% of all liver disease-related fatalities (Asrani et al., 2019).

Liver cirrhosis is a progressive complication that arises from liver disease, representing a significant advancement in hepatic fibrosis whereby there is a substantial loss of liver cells accompanied by irreversible scarring. Various factors such as viral infections (HBV and HCV), hepatic lipid accumulation, alcohol consumption, and drug toxicity contribute to chronic damage, impairing the functionality of hepatocytes. This, in turn, triggers inflammation and release of inflammatory factors, which promote excessive accumulation of collagen and extracellular matrix (ECM), resulting in the disruption of liver structure and function. Ultimately, this fibrotic process may progress to clinically significant cirrhosis and subsequent hepatic failure. Cirrhosis can be identified as an advanced stage of fibrosis characterized by the development of regenerative nodules within the liver parenchyma, enclosed by fibrotic septa (Pinzani and Macias-Barragan, 2010).

**FIGURE 4:**
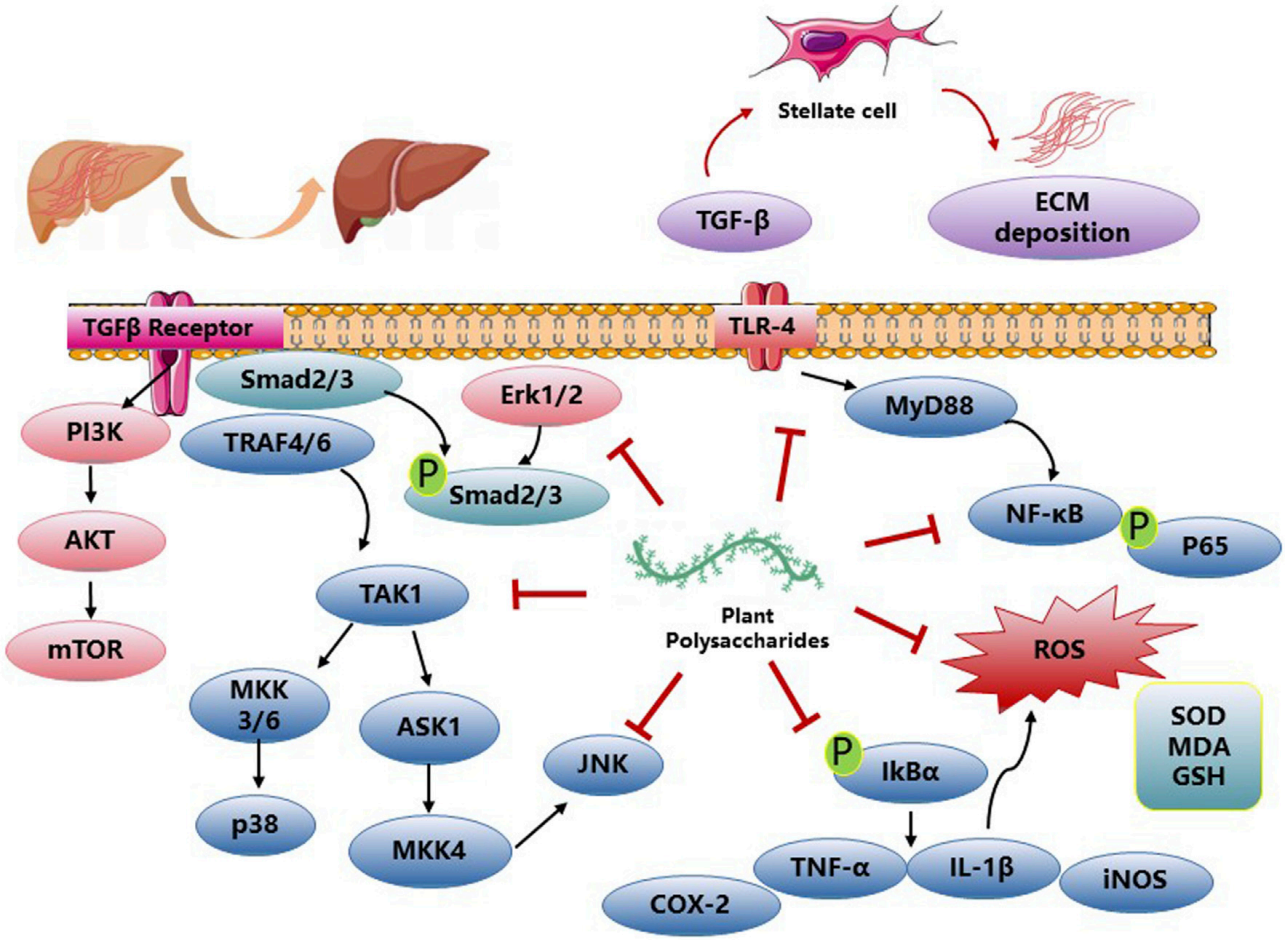
PP impact on hepatic fibrosis activities and related mechanisms.

NAFLD and ALD, as well as DILI, contribute significantly to the development of advanced liver conditions, including hepatic fibrosis and HCC. PP have been reported to exhibit a variety of pharmacological effects such as antioxidation, anti-inflammation, and anti-apoptosis, thereby improving hepatic fibrosis. The polysaccharide derived from *Talinum triangulare* demonstrates remarkable antioxidant activities, effectively reducing the levels of AST, ALT), and MDA in CCl_4_-induced liver injuries. Furthermore, it restores the activities of key antioxidant substances, SOD, and reduced GSH, thereby normalizing the liver’s defense mechanisms (Liang et al., 2011). *Lycium barbarum* polysaccharides have demonstrated effectiveness in reducing hepatic necrosis, serum ALT levels, and cytochrome P450 2E1 expression. Additionally, they restore the expression levels of antioxidant enzymes, decrease nitric oxide levels, inhibit lipid peroxidation, and alleviate hepatic inflammation. These effects are achieved through the downregulation of NFκB activity induced by CCl_4_ (Xiao et al., 2012). *Amomum villosum* polysaccharides exhibited potent *in vitro* free radical scavenging activities and effectively mitigated oxidative stress-induced liver injury in CCl_4_-treated mice by suppressing malondialdehyde formation and enhancing the activities of antioxidant enzymes (Zhang et al., 2013). Seabuckthorn berry polysaccharide (SP) administration significantly ameliorated liver injury in CCl_4_-challenged mice, as evidenced by reduced levels of serum ALT, AST, and total bilirubin (TBIL). Moreover, SP treatment enhanced PALB levels, indicating hepatoprotective effects. These beneficial effects were accompanied by increased activities of antioxidant enzymes SOD and GSH-Px, elevated GSH levels, and reduced MDA content, indicating reduced oxidative stress. SP pre-treatment also attenuated the expression of pro-inflammatory cytokines TNF-α and IL-1β, as well as iNOS and NO production induced by CCl_4_. Furthermore, SP pre-treatment suppressed hepatic TLR4 expression and inhibited the phosphorylation of p38 MAPK, p-ERK, p-JNK, and NF-κB signaling pathways in CCl_4_-challenged mice (Zhang et al., 2017). *Stipa parviflora* polysaccharides treated rat liver anti-oxidant parameters (SOD, CAT and GPx) were significantly antagonized for the pro-oxidant effect of CCl_4_ (Bargougui et al., 2019). *Dictyophora* polysaccharides (DIP) attenuated liver fibrosis induced by arsenic (As) by reducing hepatic pathological alterations and modulating the levels of serum markers including AST, ALT, total protein (TP), albumin (ALB), and Albumin/Globulin (A/G) ratio, as well as diminishing the concentrations of hyaluronic acid (HA), laminin (LN), procollagen type III (PCIII), collagen type Ⅳ (IV-C), TBIL, and direct bilirubin (DBIL). Moreover, DIP exhibited inhibitory effects on the synthesis of TGF-β1, thereby regulating the expression of connective tissue growth factor (CTGF) and subsequently suppressing the proliferation of fibrinogen and fibroblasts. This led to a reduction in fibroblast transformation into myofibroblasts, thus limiting the synthesis of fibroblasts (Wang et al., 2022). Results of the effects of PP on hepatic fibrosis activity are described in Figure 4 and Table 4.

**TABLE 4:** Summary of articles data about PP impact on hepatic fibrosis activity.

Polysaccharide	Origin plant	Models	Functions	Mechanisms	References
*Talinum triangulare* polysaccharides	*Talinum triangulare*	CCl_4_-induced liver injury in mice	Antioxidant	Decrease AST, ALT and MDA; restored antioxidant; substance SOD and GSH	Liang et al. (2011)
*Lycium barbarum* polysaccharide	*Lycium barbarum*	CCl_4_-induced acute hepatotoxicity in mice	Reduce necroinflammation and oxidative stress	Inhibit cytochrome P450 2E1 and restore antioxidant enzymes; decreased nitric oxide metabolism and lipid peroxidation	Xiao et al. (2012)
*Amomum villosum* polysaccharides	The seeds of *Amomum villosum*	CCl_4_-induced liver injury mice	Antioxidant	Prevent the formation of malondialdehyde and enhanced antioxidant enzymes	Zhang et al. (2013)
Tea polysaccharides	Green tea	CCl_4_-induced hepatotoxicity in mice	Antioxidant activity	Against free radicals	Wang et al. (2016)
*Angelica and Astragalus* polysaccharide	*Angelica and Astragalus*	CCl_4_-induced liver injury in mice	Anti-inflammation antioxidant	Ameliorate oxidative stress and to inhibit lipid peroxidation	Pu et al. (2015)
*Anoectochilus roxburghii* polysaccharides	*Anoectochilus roxburghii*	CCl_4_-induced liver injury in mice	Antioxidant	Decrease the oxidative stress marker MDA and the antioxidant enzymes	Zeng et al. (2016)
Seabuckthorn berry polysaccharide	Seabuckthorn	CCl_4_-induced liver injury mice	Anti-oxidative and anti-inflammatory	Decrease hepatic TLR4 expression and inhibit the p-p38 p-MAPK, p-ERK, p-JNK and NF-κB	Zhang et al. (2017)
Inulin-type fructan	*Artemisia vulgaris* L.	CCl_4_-induced liver injury in mice	Hepatoprotective and antioxidant	Modulate hepatic cytokines and promote a reparative inflammatory response	Correa-Ferreira et al. (2017)
*Stipa parviflora* polysaccharides	*Stipa parviflora*	CCl_4_-induced liver injury in rats	Antioxidant	Anti-oxidant parameters (SOD, CAT and GPx), hepatoprotective activity	Bargougui et al. (2019)
Radix *Cyathulae officinalis* Kuan polysaccharide	Radix *Cyathulae officinalis* Kuan	CCl_4_-induced liver injury in mice	Antioxidant	Increase antioxidant enzyme activities; decrease the production of inflammatory	Meng et al. (2019)
*Anoectochilus roxburghii* polysaccharide	*Anoectochilus roxburghii*	CCl_4_-induced liver injury in mice	Mitigate hepatotoxicity	Lipid metabolism, gut bacteria metabolism, and the methylation pathway	Zeng et al. (2020)
*Dictyophora* polysaccharides	*Dictyophora*	As-induced liver fibrosis in rats	Improve the As-induced liver fibrosis	Inhibit the synthesis of TGF-β1	Wang et al. (2022)
Pectic polysaccharide	*Abelmoschus esculentus* (Linn.) Moench	CCl_4_-induced liver injury mice	Ameliorate lipid metabolism, oxidative stress, anti-inflammatory	Regulate intestinal microflora, and promoting SCFA production	Yan et al. (2023)

#### 2.1.5 Plant polysaccharides against hepatocellular carcinoma (HCC)

**FIGURE 5:**
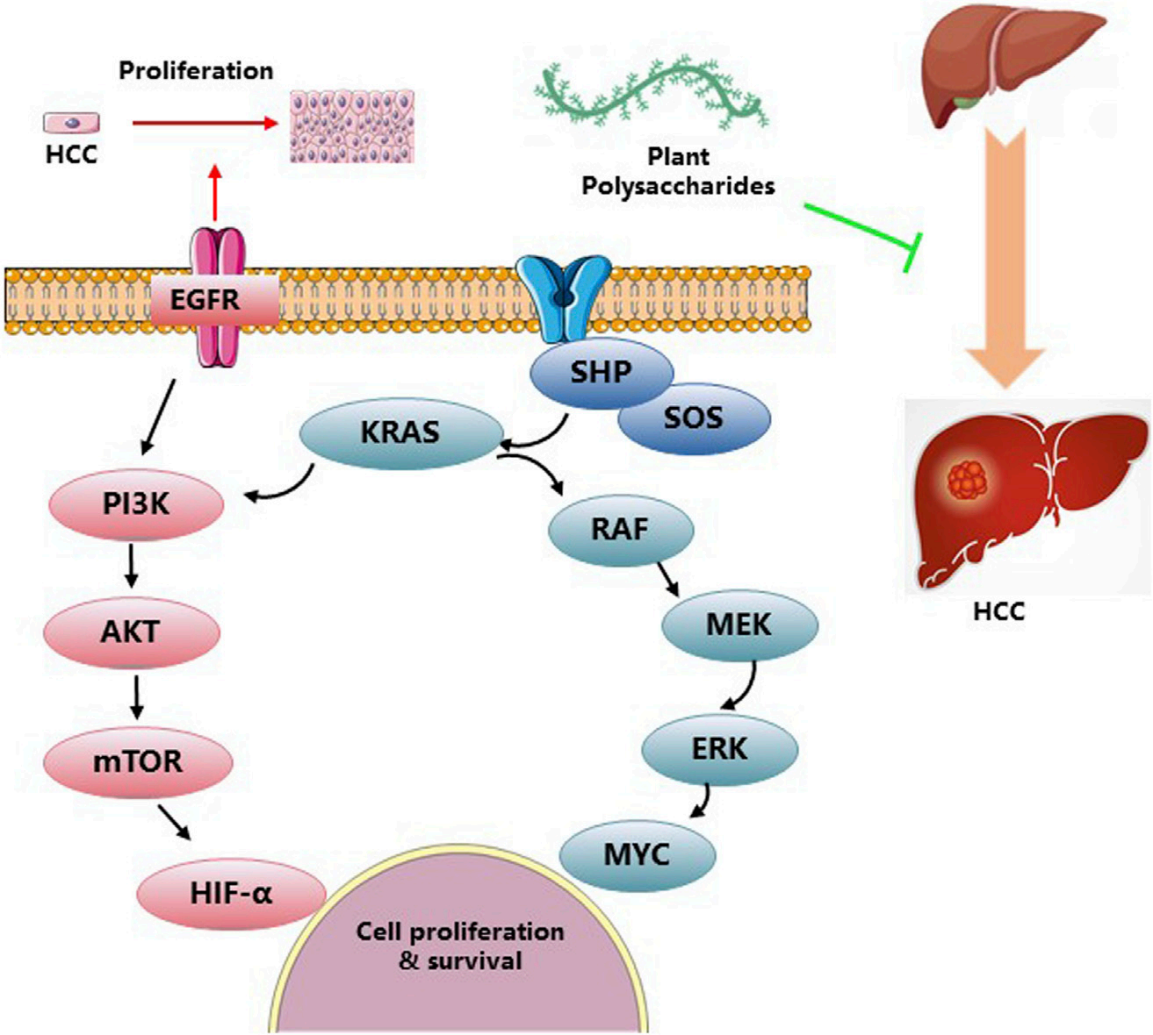
Anti-tumor activities and related mechanisms of PP.

HCC is a prevalent form of primary liver cancer with significant medical implications. It ranks sixth among the most commonly diagnosed tumors worldwide, accounting for 1.100 cases per 100,000 person-years. Moreover, it stands as the third leading cause of cancer-related mortality, resulting in 0.746 million new cases and 0.2012 million deaths. Notably, HCC represents the primary cause of death in individuals with cirrhosis, and its incidence is projected to rise in the coming years (Forner et al., 2018). The incidence of HCC is highest in East Asia and Africa, but there is a growing trend in the United States. In Asia and Africa, 60% of HCC cases are attributed to HBV infection, whereas HCV infection takes predominance in North America, Europe, and Japan. The strongest risk factor for HCC is cirrhosis, with an annual incidence ranging from 1% to 6%. HCC is commonly observed in patients with cirrhosis and is a leading cause of mortality in this population. Alcohol-induced cirrhosis accounts for 15%–30% of HCC cases, varying across geographical regions (Llovet et al., 2021). The polysaccharide derived from *Panax notoginseng* has demonstrated its potential to extend the lifespan of tumor-bearing mice by enhancing the host immune system while displaying limited cytotoxicity against hepatocellular carcinoma (Liu et al., 2021). Treatment with *Dictyophora* polysaccharides resulted in a time- and dose-dependent inhibition of HCC-LM3 cell proliferation, accompanied by cell cycle arrest in the G_2_/M phase. Moreover, the expression of Bax and caspase-3 exhibited a significant increase following *Dictyophora* polysaccharides administration (Hu et al., 2020). Dandelion polysaccharide (DP) treatment effectively suppressed the protein levels of crucial angiogenesis-related factors involved in HCC, including HIF-1α, VEGF, p-PI3K, and p-AKT. This suggests that DP holds promise as a potential therapeutic agent for HCC (Ren et al., 2020). Astragalus polysaccharide (APS) has been found to mitigate PD-L1-mediated immunosuppression by targeting the miR-133a-3p/MSN axis, thereby facilitating an antitumor response (He et al., 2022). Additionally, ASP has been explored as a targeted drug carrier for HepG2 tumors via ASGPR, enhancing therapy for liver cancer (Zhang et al., 2019). Treatment with *Aconitum coreanum* polysaccharide, a potential therapeutic agent for HCC, led to a significant reduction in p-Akt protein levels, while simultaneously increasing p-p38 MAPK protein levels in H22 cells (Liang et al., 2015). Hypoxia promotes epithelial-mesenchymal transition (EMT) and facilitates migration and invasion of HCC cells. However, Basil polysaccharide (BPS) exhibits inhibitory effects on tumor progression and metastasis, including the reversal of EMT via cytoskeletal remodeling under hypoxic conditions. Furthermore, BPS targets hypoxia-inducible factor 1 alpha (HIF1α) to alleviate tumor hypoxia. We observed downregulation of mesenchymal markers (β-catenin, N-cadherin, and vimentin) along with upregulation of epithelial markers (E-cadherin, VMP1, and ZO-1) after BPS treatment, highlighting its potential for HCC therapy in hypoxic conditions (Feng et al., 2019). The application of *Ginseng polysaccharide* (GSP) mainly includes GSP injection and GSP fermented milk beverage. GSP injection is mainly used as an adjuvant therapy for clinical tumors, reducing the side effects caused by various tumor radiotherapy and chemotherapy, and serving as an immune modulator to improve the immune function. It can also be used to treat acute and chronic hepatitis and various liver injuries, as well as various chronic infections, diabetes, and various immune diseases. Figure 5 and Table 5 give a summary of a few reports on the anti-tumor activity of PP.

**TABLE 5:** Summary of known anti-tumor activity of PP.

Polysaccharide	Origin plant	Models	Functions	Mechanisms	References
*Andrographis paniculate* polysaccharide	*Andrographis paniculate*	HepG2 cells	Loss of mitochondrial membrane potential and the release of cytochrome c from the mitochondria to the cytosol; caspase-9 and caspase-3 were activated	Mitochondria-mediated signaling pathway	Zou et al. (2015)
*Aconitum coreanum* polysaccharide	*Aconitum coreanum*	H22 cells in mice	The induction of apoptosis	PTTG1-mediated suppression of the P13K/Akt and activation of p38 MAPK signaling pathway	Liang et al. (2015)
Purified white polysaccharide	Purified white	HepG2 cells	Apoptosis	Induce apoptosis involved a caspase-3-mediated mitochondrial pathway	Shen et al. (2016)
*Dandelion* Polysaccharide	*Dandelion*	Hepa1-6 cells; H22 cells tumors model	Suppress expression of VEGF and HIF-1α	PI3K/AKT signaling	Ren et al. (2020)
*Dictyophora* polysaccharide	*Dictyophora*	HCC-LM3 cell line	Modulate Cell cycle and apoptosis-inhibited cell	Proliferation in a time- and dose-dependent manner and block the cell cycle in the G₂/M phase	Hu et al. (2020)
crude Polysaccharide	*Panax notoginseng*	myelosuppression mice induced by CTX	Antitumor activity for the treatment of liver cancer combined with cyclophosphamide	Immunosuppressive	Liu et al. (2021)
*Astragalus* polysaccharide	*Astragalus*	Hep3B xenograft tumors model	Inhibit exacerbation of ER stress and activation of apoptotic	Promote Dox-induced apoptosis through reducing the O-GlcNAcylation; mediated immunosuppression via miR-133a-3p/MSN axis; decreasing the expression of Notch1	He et al. (2022), Huang et al. (2019), Li et al. (2023)
Mushroom-derived polysaccharide	Mushroom	N-Diethylnitrosamine-Induced Hepatocellular Carcinoma in Wistar Rats	Antioxidant and anti-inflammatory; exerted cytotoxic activities	Inhibit cell proliferation and restore liver architecture, antioxidant enzymes, and cytokines/chemokines balance	Sipping et al. (2022)

## 3 Conclusion and outlook

Chronic liver disease has a significant impact on global health, resulting in over two million deaths annually and accounting for 4% of all global deaths (Devarbhavi et al., 2023). However, there are limited hepatoprotective drugs available in the market that demonstrate satisfactory efficacy without notable side effects. Natural compounds, characterized by their structural diversity and beneficial biological activities, hold great potential as precursors for clinical drugs. Therefore, the search for natural and low-toxic hepatoprotective compounds is of utmost importance. Plant polysaccharides (PP) from herbs have emerged as active constituents with a wide range of pharmacological effects, including antioxidant, anti-inflammatory, anti-apoptotic, lipid metabolism regulation, and anti-cancer properties. PP have demonstrated promising hepatoprotective effects against various liver conditions such as NAFLD, ALD, DILI, hepatic fibrosis and HCC.

In addition, cholestatic hepatitis is a disorder characterized by aberrant metabolism of bile acids. However, there is limited research on the role of polysaccharides in cholestatic hepatitis. Although polysaccharides with pharmacological effects, including antioxidant, anti-inflammatory, anti-apoptotic, which may have a therapeutic effect like *Yinchenhao decoction* (YCHD). YCHD exhibited the ability to ameliorate cholestasis by stimulating the bile secretion pathway. The YCHD has been proposed for its potential to mitigate cholestasis by activating the bile secretion pathway. This mechanism entails the modulation of various targets, including FXR, by some of the major active components of YCHD. FXR, a vital molecule that interacts with bile acids, exerts inhibitory effects on bile acid synthesis and transport (Keitel et al., 2019). FXR modulates the expression of the bile salt export pump (BSEP), facilitating the translocation of monovalent bile acids from hepatocytes to bile, thereby mitigating the hepatotoxicity associated with bile acids (Luo et al., 2024). In conclusion, this review provides a comprehensive overview of the latest advancements regarding PP and their significance in modulating lipid metabolism, inflammation, fibrosis, and oxidative stress. These effects are predominantly regulated through classical signaling pathways, such as MAPK, AMPK, PPAR, NFκB, and PI3K/AKT, which play crucial roles in the development and progression of diverse liver disorders.

PP have shown great potential for clinical use due to their remarkable properties. However, despite the numerous advantages revealed by several clinical trials, PP utilization in real-world medical settings remains limited. While their safety, effectiveness, and minimal side effects have been verified (Yang et al., 2022; Kuang et al., 2023), unresolved issues persist. For example, the health benefits and medicinal value of *Ganoderma lucidum* polysaccharide (GLP) have been widely recognized in the academic community. Although GLP has not yet been used in clinical medicine, it will have broad application prospects and clinical value as its immunomodulatory, tumor suppressive, and blood glucose regulating effects and mechanisms are gradually elucidated. At present, there are still some controversies regarding the widespread application of GLP: firstly, the composition of GLP is complex, and different types and origins of Ganoderma have an impact on the composition of GLP; Secondly, the molecular regulatory mechanisms of GLP in inhibiting tumors and lowering blood sugar are not fully understood and lack in-depth research; Thirdly, research on the efficacy of GLP is still in the experimental stage, with limited clinical research. It is still necessary to study and explore how to determine the clinical value and effective dosage of GLP. The biological activities of PP are strongly impacted by their chemical structure and chain conformations, necessitating further investigation and improved methodologies to establish their efficacy. Two crucial areas for future research can be identified. First and foremost, it is crucial to enhance the bioavailability of plant polysaccharides (PP) and identify their specific molecular targets for the treatment of chronic liver disease (CLD). These factors are essential for advancing research and developing new PP-based drugs in the future. Additionally, with accurate knowledge of the structure and molecular weight of PP, extensive studies have investigated the structure-activity relationship of PP in CLD treatment. However, further investigations are required to attain rational structural optimization and design of PP, thereby influencing critical signaling pathways involved in multifactorial liver diseases to enhance bioavailability and therapeutic efficacy. This research will facilitate a deeper understanding of the precise binding sites and mechanisms through which these polysaccharides operate. This foundational knowledge will pave the way for further research and the potential development of innovative medications. Lastly, it is imperative to conduct clinical trials to evaluate the safety and effectiveness of PP in the management of multifactorial liver disease. Furthermore, the combination of PP with other drugs for comprehensive treatment has emerged as a recent research focus.

There is a general conception that PP, when utilized as health food or dietary supplements are generally considered safe to enhance immune response, exhibit potential antiviral effects, antioxidant properties, modulation of inflammatory pathways and potential anti-cancer properties. For example, Serum glucose was found to be significantly decreased and insulinogenic index increased during OMTT after 3 months administration of *Lycium barbarum* polysaccharide. In addition, a prescription drug, injectable *Astragalus* polysaccharide (PG2) (PhytoHealth Corporation, Taiwan, ROC), is an immunomodulator that has been approved for the alleviation of cancer-related fatigue. However, they can cause adverse effects when used therapeutically, such as in the examples shown in Table 6. The combined use of PP with other drugs are considered a major problem in clinical practice of PP as they contribute to altering important pharmacokinetic processes, resulting in therapeutic failure or increase toxicity of some prescription drugs. Clinical trials have shown that many over-the-counter dietary supplements can modulate the activity of drug metabolizing enzymes and/or drug transporters and further influence the bioavailability of co-administrated drugs (Abuznait et al., 2011). For example, *Lycium barbarum* polysaccharide could inhibit cytochrome P450 (Xiao et al., 2012), yet herb-drug and food-drug interactions can arise from the modulation of cytochrome P450 isoenzymes. Therefore, the adverse effects of synthetic action of PP with other drugs for comprehensive treatment should be considered in the treatment of patients with chronic liver disease whose liver function may already be compromised. Additionally, a significant challenge in harnessing the full potential of these polysaccharides is their limited solubility, which hampers the development of effective dosage forms. To overcome this limitation, the utilization of nano-formulations has emerged as a promising approach, improving the bioavailability of these compounds. Moving forward, the ongoing advancements in PP research offer great potential for revolutionizing treatment strategies for CLDs.

**TABLE 6:** Summary of main findings and side effects of PP in clinicals.

Polysaccharide	Functions	Side effects	References
*Lycium barbarum* polysaccharide	Improved immune function, antioxidant properties, and potential anti-inflammatory effects	Generally well-tolerated. Some individuals may experience mild gastrointestinal discomfort	Cai et al. (2015), Chen et al. (2020), Gao et al. (2021)
*Astragalus* polysaccharide	Enhanced immune response, potential antiviral effects, and modulation of inflammatory pathways	Rare cases of allergic reactions or gastrointestinal upset have been reported	Bamodu et al. (2019), Huang et al. (2019), Tsao et al. (2021), Lee et al. (2023)
*Ganoderma lucidum* polysaccharide (fungal polysaccharides)	Exhibits immunomodulatory effects, potential anti-cancer properties, and antioxidant activity	Rare cases of liver toxicity or allergic reactions have been reported with high doses	Lin et al. (2005), Lin et al. (2006)

## Abbreviations

AMPK, adenosine 5′-monophosphate (AMP)-activated protein kinase; ALD, alcohol-related liver disease; CLD, Chronic liver disease; DILI, drug-induced liver injury; LPS, lipopolysaccharide; NAFLD, non-alcoholic fatty liver disease; PPAR-α, peroxisome proliferator-activated receptor-α; PP, Plant Polysaccharides; SREBP-1, sterol-regulatory element binding proteins; ZO-1, zonula occludens-1.
